# Fudging the volcano-plot without dredging the data

**DOI:** 10.1038/s41467-024-45834-7

**Published:** 2024-02-15

**Authors:** Thomas Burger

**Affiliations:** Univ. Grenoble Alpes, INSERM, CEA, UA13 BGE, CNRS, CEA, FR2048 ProFI, 38000 Grenoble, France

**Keywords:** Data processing, Proteome informatics, Statistics, Proteomics, Statistical methods

## Abstract

Selecting omic biomarkers using both their effect size and their differential status significance (*i.e.*, selecting the “volcano-plot outer spray”) has long been equally biologically relevant and statistically troublesome. However, recent proposals are paving the way to resolving this dilemma.

In their recent *Nature Communications* article, Bayer et al. present the tool CurveCurator^1^ to select biomarkers according to their dose-response profiles, with well-established statistical guarantees. To conveniently blend the effect size and the significance of the dose-response curve into a single *relevance score*, they revisit the so-called fudge factor introduced in the SAM test^2^. Moreover, to overcome the risk of involuntary data dredging inherent to “fudging” the differential analysis^3^, they propose a new approach inspired by the target-decoy competition framework (TDC^4^). The principle of TDC is to add counterfactual amino acid sequences (termed decoys) to a (target) database of real amino acid sequences, as to mimic erroneous matches in a peptide identification task. Despite its original empirical-only justifications (peptide matches involving decoy sequences should be as probable as mismatches involving target sequences), TDC has long been used in mass spectrometry-based proteomics to validate peptide identifications according to a False Discovery Rate (FDR^5^) threshold. Accordingly, Bayer et al. claim FDR control guarantees regardless of the fudge factor tuning. Several recent works in selective inference (a subfield of high-dimensional statistics) have provided theoretical support to their intuition^6,7^, which justify its generalization to a variety of similar situations. Concretely, this comment asserts that essentially any omics data analysis involving a volcano-plot is concerned –be it transcriptomics, metabolomics, proteomics or any other; either at bulk or single cell resolution. Therefore, elaborating on Bayer et al. visionary proposal should lead to new user-tailored computational omic tools, with sweeping consequences from the application standpoint.

## Issues pertaining to the fudge factor

**Fig. 1:**
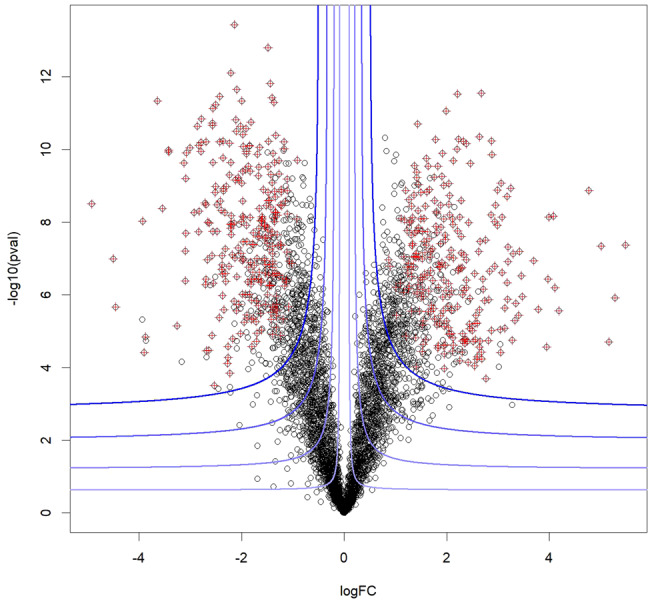
A typical volcano-plot. A significance measure is depicted on the Y-axis (here, -log10(p-value)) and an effect size is depicted on the X-axis (here, the logarithmized fold-change). The blue lines represent the contours of the relevance score and the points highlighted in red are those selected according to a knockoff procedure.

While the fudge factor was originally introduced as a small positive constant (denoted as ${s}_{0}$) to improve the independence of the test statistic variance and of the omic feature expression, its tuning to a larger value has been observed to yield a user-defined weighting of the significance and of the effect size. Concomitantly, the permutation-based procedure of SAM test has sometimes been replaced by classical p-value adjustment –as prescribed in the Benjamini-Hochberg (BH) procedure for FDR control^5^. Applying simultaneously these two tricks enhances volcano-plot interpretation: the biomarkers selected are located in the outer spray of the volcano-plot, with selection boundaries following hyperbolic contours (see Fig. 1). Unfortunately doing so jeopardizes the statistical guarantees: briefly, a too large ${s}_{0}$ value distorts the p-values as well as the subsequent adjusted p-values calculated in the BH procedure. To cope with this, it is either necessary to constrain the tuning of ${s}_{0}$ (at the cost of less flexible selection of the outer spray) or to replace BH procedure by another FDR control method that does not require any p-value adjustment. Although the permutation-based procedure associated to SAM test is an option, it does not strictly controls for the FDR (see Table 1). Bayer et al. have thus explored another option inspired by TDC, which has emerged nearly twenty years ago in proteomics in absence of p-values to assess the significance of peptide identification.

**Table 1:** Pros and cons of the various approach to FDR control with respect to selecting biomarkers on the outer spray of the volcano-plot

Approach to FDR control	Advantages	Disadvantages
P-value adjustment/q-value	•Standard, easy to apply and computationally efficient.	•Requires well-calibrated p-values. •Issue with FC filtering^3,11,12^, either following hyperbolic contours or not.
Empirical Bayes/null	•Can cope for most of the drawbacks of the above methods (p-value calibration and FC interaction).	•Requires the capability to tune the priors. •Does not have frequentist interpretation, which may hinder objective significance assessment^13^.
Permutations	•The multiple test correction is non-parametric. •No calibration issue. •Related works based on FDP bounding authorize double-dipping^14^.	•Strictly speaking, does not control for the FDR; Instead, it provides a probabilistic upper bound to the FDP^15^. •The fudge factor should not be tuned in contradiction to the statistical guidelines^2^.
Knock-offs/decoys	•Flexibility of the relevance score.	•Instable w.r.t. KO generation^10^. •Difficulty of assessing the KO generation (which can lead to overly conservative FDR control).

## Competition-based alternatives to control for the FDR

Although published a decade later, the most convincing theoretical support of TDC to date has been knock-off filters (or KO)^6,7^. In spite of minor discrepancies with TDC^8^, KO mathematically justifies TDC general approach to FDR control, as well as its main computational steps. Notably, it demonstrates that FDR can be controlled on a biomarker selection task by thresholding a contrast of relevance scores, which results from a pairwise competition between the real putative biomarkers and other ones, fictionalized –respectively referred to as decoys and knock-offs in the proteomic and statistic parlances. Intuitively, the proportion of fictionalized features selected should be a decent proxy of the ratio of false discoveries [*Nota Bene*: In KO theory, this proportion is corrected by adding 1 to the ratio numerator to cope for a bias issue. Although this bias is still investigated^9^, this suggests to correct for Eq. 16 in^1^ by adding 1 to the numerator too.], as long as the decision is made symmetrically (*i.e*.﻿,﻿ their relevance score is attributed regardless of their real/fictional status). However, despite conceptual similarities, the problems solvable by TDC and KO differ: For the former, features are classically amino acid sequences; while for the latter, a quantitative dataset describing biomolecular expression levels in response to various experimental conditions is classically considered. In this context, the TDC extension proposed in CurveCurator to process quantitative dose-response curves constitutes a nice bridge between the TDC and KO kingdoms.

## Generalizing the CurveCurator approach

With this in mind, the pragmatic fallouts of Bayer et al. become striking. Any data analyst wishing to select omic biomarkers with a relevance score picturing hyperbolic contours on a volcano plot (see Fig. 1) can easily adapt CurveCurator approach to their own case, by following the above procedure:

1. Perform statistical tests to obtain a p-value for each putative biomarker that assess the significance of its differential status,
2. Likewise, compute the biomarker fold-change, as a measure of the effect size, and construct the volcano-plot,
3. Tune ${s}_{0}$ to blend the significance of the differential status and the effect size into a single relevance score,
4. Acknowledge the relevance score looks like a p-value even though it may not be valid to use it as such, depending on the ${s}_{0}$ chosen,
5. Rely on the KO framework (*e.g*., using the “knockoff” R package (https://cran.r-project.org/web/packages/knockoff/index.html) as well as on the numerous tutorials available (https://web.stanford.edu/group/candes/knockoffs/software/knockoffs/) to control for the FDR on the biomarker selected according to the relevance score, in a way similar to that of CurveCurator.

## Different FDR control frameworks for different situations

An important and possibly troublesome feature of Fig. 1 is that some “unselected” black points are surrounded by “selected” red ones. In other words, some putative biomarkers may not be retained while other ones with smaller effect size and larger raw p-value are. This is a classical drawback of competition-based FDR control methods: each putative biomarker being retained or not does not only depend on its features, but also on those of its fictionalized counterpart, which generation is subject to randomness. Although this weakness can be addressed too, it requires less straightforward tools^10^. Another still open problem in KO theory lies in the KO/decoy generation, which can be difficult depending on the dataset. With this respect, the approach of CurveCurator is worthwhile. More generally, no method is perfect: KO filters, like p-value adjustment or permutation-based control have pros and cons (see Table 1). Therefore, depending on the data analyst ‘need, the preferred method should change. Considering this need for multiple off-the-shelf tools, it is important to notice that KO filters have hardly spread beyond the theoretical community so far, and that their applications to enhance data analysis in biology-centered investigations are still scarce, unfortunately. In this context, the seminal proposal of Bayer et al. can be expected to foster the translation of these fast-evolving theories into practical and efficient software with growing importance in biomarker discoveries, and they must be acknowledged for this.
